# Clinical Outcome Discrimination in Pediatric ARDS by Chest Radiograph Severity Scoring

**DOI:** 10.1155/2022/9309611

**Published:** 2022-05-14

**Authors:** Yu-Chun Yan, Wen-Han Hao, Feng-Sen Bai, Shuang Liu, Dong Qu, Xin-Yu Yuan

**Affiliations:** ^1^Department of Radiology, The Affiliated Children's Hospital, Capital Institute of Pediatrics, Beijing, China; ^2^Department of Critical Medicine, The Affiliated Children's Hospital, Capital Institute of Pediatrics, Beijing, China

## Abstract

**Background:**

There is no accurate radiological measurement to estimate the severity of pediatrics acute respiratory distress syndrome (PARDS). We validated the effectiveness of an adult radiographic assessment of lung edema (RALE) score in PARDS.

**Aim:**

To assess the severity and prognosis of PARDS based on a chest radiograph (CXR) RALE scoring method.

**Methods:**

Pediatric Acute Lung Injury Consensus Conference (PALICC) criteria were used to diagnose PARDS. General demographics, pulmonary complications, and 28‐day mortality of the patients were recorded. Subgroups were compared by prognosis (survive and death) and etiology (infection and noninfection). Two observers calculated RALE independently. Each quadrant of CXR was scored by consolidation scores 0 (none alveolar opacity), 1 (extent <25%), 2 (extent 25%–50%), 3 (50%–75%), and 4 (>75%) and density scores 1 (hazy), 2 (moderate), and 3 (dense). Quadrant score equals consolidation score times density score. Total score equals to the sum of four quadrants scores. The ROC curve and survival curve were established, and the optimal cutoff score for discrimination prognosis was set.

**Results:**

116 PARDS (72 boys and 44 girls) and 463 CXRs were enrolled. The median age was 25 months (5 months, 60.8 months) and with a mortality of 37.9% (44/116). The agreement between two independent observers was excellent (ICC = 0.98, 95% CI: 0.97–0.99). Day 3 score was independently associated with better survival (*p* < 0.001). The area under the curve of ROC was 0.773 (95% CI: 0.709–0.838). The cutoff score was 21 (sensitivity 71.7%, specificity 76.5%), and the hazard ratio (HR) was 9.268 (95% CI: 1.257–68.320). The pulmonary complication showed an HR of 3.678 (95% CI: 1.174–11.521) for the discrimination.

**Conclusion:**

CXR RALE score can be used in PARDS for discriminating the prognosis and has a better agreement among radiologist and pediatrician. PARDS with pulmonary complications, day 3 score whether greater than 21 points, have a better predictive effectiveness.

## 1. Introduction

Acute respiratory distress syndrome (ARDS) is a complex syndrome with heterogeneous causes and diseases and carries high rates of morbidity and mortality [1, 2]. The largest PARDS validation (PARDIE study) showed the International Pediatric ARDS (PARDS) incidence was 3.2% amongst pediatric intensive care units (PICU) patients and the mortality for severe PARDS was up to 33% [3]. According to Pediatric Acute Lung Injury Consensus Conference (PALICC) PARDS definition, not only lung mechanics, oximetry, and blood gases should be noted but also the chest imaging [4]. The image pattern of PARDS can be unilateral or bilateral pulmonary infiltrates. Although image manifestations frequently lag behind the development of hypoxemia, the different distribution pattern can help choose specific ventilatory setting, monitor therapeutic response, and even predict clinical outcome [4–6].

The modality of imaging evaluation of PARDS includes chest radiograph (CXR), CT, and ultrasound. Despite CT is the gold standard to demonstrate precise morphology of lung ventilation, the safety issue for patient transfer and radiation exposure limits its utility [7]. As a radiation-free and noninvasive exam, transthoracic lung ultrasound (LUS) shows the convenience in PARDS evaluation [8, 9]. Subcutaneous emphysema, large thoracic dressings, providers' skills, and experience might limit its efficiency in particularly cases [9]. CXR remains an essential role in clinical practice.

Since the extent and degree of alveolar damage on CXR reflect the severity, Warren and colleagues established a radiographic assessment of lung edema (RALE) scoring method in adult, enriching a novel tool to predict the prognosis in ARDS [10]. After its establishment, relevant studies on adults were published [11, 12]. However, to our knowledge, the study of RALE score validation on children is still rare. Herein, the study aimed to assess the severity and prognosis of the children who met the criteria of PARDS. Furthermore, compare the consistency utilized by radiologist and pediatrician, investigate the relations with CXR and severity, and discriminate the prognosis based on the RALE scoring method.

## 2. Materials and Methods

### 2.1. Study Design

This study was a single‐center retrospective observational study in nature between January 1^st^ 2018 to June 30^th^ 2021. Institution ethical committee approval (KSSHERLL2018005) was taken prior to commencement of study. The informed consent was obtained.

### 2.2. Participant Recruitment

Patients admitted to PICU were eligible for the study if they met PALICC PARDS diagnostic criteria, received strictly invasive mechanical ventilation (IMV), had bedside CXR exams, and etiology of pulmonary infections proven by sample culture and/or DNA quantitative polymerase chain reaction (PCR) testing (bacterial/viral/fungal). The exclusion criteria included age ≤28 days old, admission time less than 24 h, received extracorporeal membrane oxygenation (ECMO) therapy, special populations, namely, patients with cyanotic heart disease, chronic lung disease, and left-ventricular dysfunction, and incomplete clinical or CXR data.

### 2.3. Data Collection

Patient's data were recorded and compared as follows: general demographics, including age, sex, etiology of ARDS, intubation time, oxygenation index (OI), positive end-expiratory pressure (PEEP), and SpO_2_; number of performed CXRs and CXR RALE scores; pulmonary complications, namely, air-leak syndrome, pleural effusion, and alveolar hemorrhage; and prognosis, 28‐day mortality. Subgroups were divided according to the prognosis (survival and death). Infection and pulmonary complications were recorded as risk factors to compare for the discrimination.

### 2.4. CXR RALE Scoring

**Figure 1:**
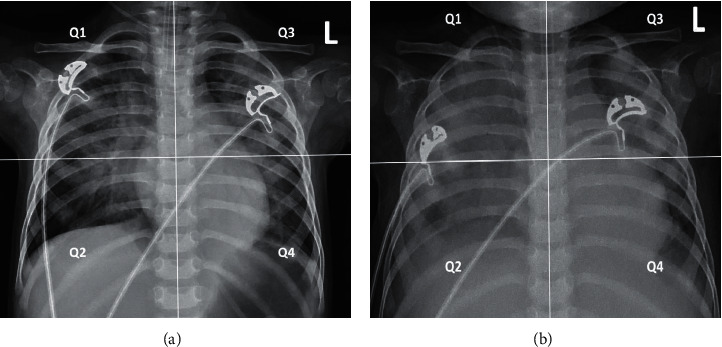
RALE scoring of a 19-month-old girl died of H1N1 pneumonia. (a). Day 1 (since intubation) RALE score was 16 points (*Q*1 = 2 × 2, *Q*2 = 2 × 2, *Q*3 = 2 × 2, *Q*4 = 2 × 2). (b). Day 3 RALE score was 27 points (*Q*1 = 4 × 2, *Q*2 = 4 × 2, *Q*3 = 3 × 1, *Q*4 = 4 × 2). ^*∗*^Consolidation scores 0 (none alveolar opacity), 1 (extent <25%), 2 (extent 25%–50%), 3 (50%–75%), and 4 (>75%). Density scores 1 (hazy), 2 (moderate), and 3 (dense).

Each CXR was divided into four quadrants, vertically by the midline of spine and horizontally at the level of left upper and lingual lobe bronchus (first branch of the left main bronchus). Based on the RALE score, the extent (consolidation score) and degree (density score) of each quadrant will be calculated, respectively, as follows [10]: consolidation scores 0 (none alveolar opacity), 1 (extent <25%), 2 (extent 25%–50%), 3 (50%–75%), and 4 (>75%) and density scores 1 (hazy), 2 (moderate), and 3 (dense). Quadrant score equals consolidation score times density score. Total score equals to the sum of four quadrants scores, as shown in Figures 1(a) and 1(b). According to PALICC PARDS criteria, patients with unilateral pulmonary infiltrate were also subjected to the RALE method.

Each CXR was scored independently by two observers (a radiologist with 17 years' experience and an advanced pediatrician with 14 years' experience), in order to evaluate interobserver variation. Define day 1 (since intubation) as baseline. If multiple CXRs were performed in a single day, the most severe one for scoring was selected by the observers.

### 2.5. Statistics

All statistical analyses were performed with Jeffrey's Amazing Statistics Program (JASP, version 0.14.1). All continuous variables that conform to the normal distribution were expressed by mean ± standard deviation ($\bar{x}\pm s$). Variables with an abnormal distribution were described with the median value (median, interquartile range, 25–75%). We used the two-way random model (absolute agreement type) to calculate intraclass correlation coefficient (ICC) to assess the reliability of two independent observers. Bland–Altman plots were used to show the agreement of independent observers. The chi-square test was used to compare sex, infection, and pulmonary complication. The *t*-test was to compare age, intubation time, OI, PEEP, SpO_2_, and RALE scores. Receiver operating characteristic curve (ROC) analysis was performed, and the area under the ROC curve (AUC) was calculated. Cox regression (which was based on the proportional-hazards model) was used to calculate the risks. The level of significance was set to 0.05.

## 3. Results

### 3.1. Comparisons of PARDS

**Figure 2:**
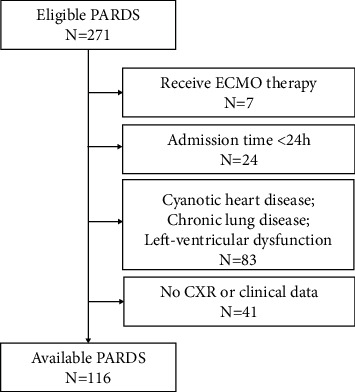
Selection process of patients. 116 patients of the 271 were enrolled. ECMO, extracorporeal membrane oxygenation; CXR, chest X-ray.

Finally, 116 patients of the 271 had matched the above criteria, and a total of 463 CXRs were performed (Figure 2). The median age of 116 PARDS patients was 25 months (5 months, 60.8 months), 72 boys and 44 girls. The mortality was 37.9% (44/116). Among them, 56.0% (65/116) were infection patients (virus *n* = 37, bacteria *n* = 23, and fungus *n* = 5), and 31.0% (36/116) had pulmonary complications (air-leak syndrome *n* = 14, pleural effusion *n* = 18, and alveolar hemorrhage *n* = 4). Characteristics of 116 patients are given in Table 1 and Table 2. OI score, PEEP, and SpO_2_ showed a statistically significant difference in the survival/death and infection/noninfection groups. Pulmonary complications were commonly seen in the death group (*χ*^2^ = 11.913, *p* < 0.001). There was no statistically significant difference in age, sex, and intubation time between two groups.

### 3.2. Validation of RALE Score in PARDS

**Figure 3:**
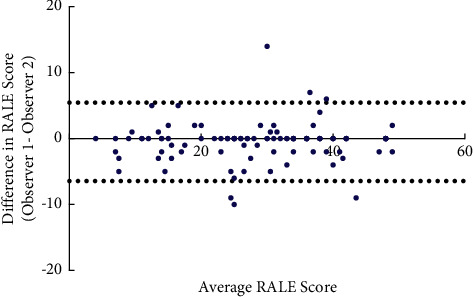
Bland–Altman plots showing agreement of two independent observers of RALE scores.

The scores of two observers were compared, the ICCs were excellent (ICC = 0.98, 95% CI: 0.97–0.99), and Bland–Alman plots also showed a better agreement between two independent observers of RALE scores (bias = −0.49, SD of bias = 3.035, 95% CI of limits of agreement: −6.44–5.45) (Figure 3).

**Figure 4:**
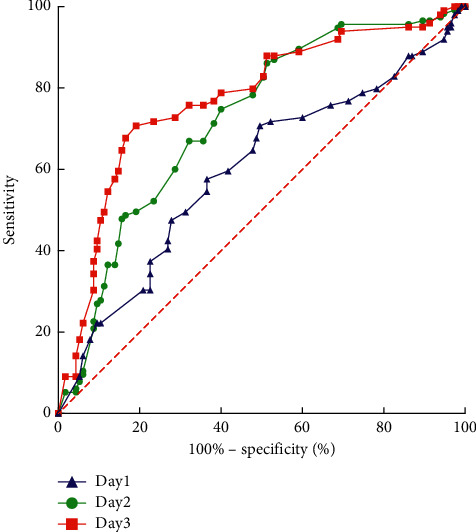
ROC curve of RALE score from day 1 to day 3. The area under the curve of day 3 was 0.773 (95% CI: 0.709–0.838), higher than day 1 and day 2.

**Figure 5:**
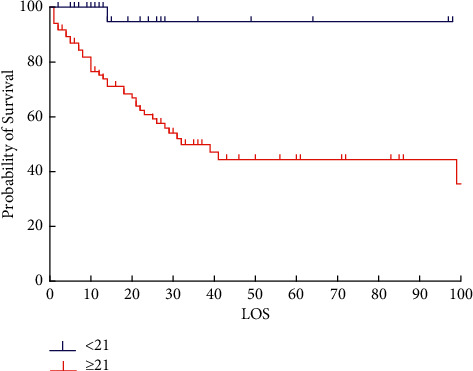
Survival curve of day 3 by score of 21.

The RALE score of the survival group declined since day 1, whereas the RALE score of the death group had a peak on day 3 (*t* = −6.248, *p* < 0.001). Compared to day 1, the RALE score of day 3 was independently associated with survival. The ROC showed the area under the curve for predicting was 0.773 (*p* < 0.001, 95% CI: 0.709–0.838) (Figure 4). Set the cutoff score at 21, the sensitivity was 71.7%, while the specificity was 76.5%, and hazard ratio (HR) was 9.268 (95% CI: 1.257–68.320). The survival curves showed that RALE score lower than 21 at day 3 had better survival (Figure 5). The pulmonary complication showed an HR of 3.678 (*p* < 0.001, 95% CI: 1.174–11.521) for the discrimination. In infection PARDS patients, day 3 RALE score was significant different than that of day 1 (*t* = −6.178, *p* < 0.001) (Table 3).

## 4. Discussion

The main objective of this study was to validate whether the novel chest radiograph scoring method applied in adults for evaluating lung edema was also applicable in pediatric ARDS patients. The CXR RALE score in children was also well correlated with overall disease severity and could predict clinical outcomes. As a marker for clinical prognosis, this practical simple bedside tool reinforces clinical management since it is easy to interpret and assess through the basic clinical imaging modality. The mortality rate of ARDS in adults and severe PARDS is basically the same, and the resources required and costs of care are significant due to the severity [3, 13–15]. Even though, an efficient quantitative score may allow predict clinical course and help to improve management.

Warren and her colleagues established the RALE score to evaluate lung edema, which considered the extent and density to reflect ARDS severity [10]. Although the original intention of RALE was to evaluate lung edema, this pathological change was the key feature in ARDS [16]. According to PALICC diagnostic criteria, pulmonary edema was not fully explained by heat failure or fluid overload [4]. The common methods for pulmonary edema evaluation are either invasive (catheter) or difficult to performance (computed tomographic quantitative imaging). Both methods should concern safety issue. At present, pulmonary ultrasound plays an important role to reduce X-ray exposure especially in infants [17]. Even though CXR remains undisputable, it can demonstrate an overview of pulmonary and cardiovascular condition, which is better than LUS. Both pulmonary and hypoxemia (impaired oxygenation) are reflected on CXR to some extent. Thus, correlating their relations can provide a novel idea for clinical evaluation of disease severity. Recently, Raissaki and her colleagues revised a 5-point scale score for assessing the severity of acute respiratory failure [6]. Beyond that, to our knowledge, this is the first study that used the RALE score based on CXR for PARDS and correlated well with clinical discrimination.

In our cohort, the mortality was 37.9%, much higher than the PARDIE study [3]. The reason for the disparity was that the patients who had incomplete clinical or CXR data had just been excluded. The RALE score showed different trends in the death group than in the survival group and represented that the severe PARDS was progressing faster in clinical course [18]. This is because the spectrum of diseases in children is different from that in adults. In this study, 56.0% (65/116) patients were bacterial and virus infection, while cardiopulmonary chronic diseases were commonly combined in the elders and trauma in young adults [19]. Pulmonary complication was a significant risk factor in predicting prognosis, which showed an HR of 3.678 (95% CI: 1.174–11.521) for death, while pleural effusion was more in infection disease and air-leak was common in noninfections.

We tried to find the trend of the PARDS course, set day 1 (since intubation) RALE score as baseline, the ROC curve showed a significant difference in day 3 RALE score, and the AUC was 0.773 (95% CI: 0.709–0.838). Combined with 21 points as the cutoff value showed statistical significance (*p* < 0.001), the sensitivity was 71.7%, while the specificity was 76.5%, and HR was 9.268 (95% CI: 1.257–68.320). The above indicators can be early warning to the clinician. After day 3, the trend of the RALE score was of great significance to the clinical prognosis. The gradual decrease of the score indicated that the disease was alleviated, and the prognosis would be good. The score continued to rise, indicating that the condition was maintained or worsened.

A recent study showed that the interpretation of CXR in PARDS varies between radiologist and physicians [20]. The ICC and Bland–Altman plots in this study showed better agreement; the reason is that the items RALE score chose to evaluate are simple and easy to quantify. Only extent and dense of the infiltration should be noticed, rather than variability of imaging findings. Thus, the RALE score is more practical. Compared to the RALE study in adult, the RALE score in severe patients were basically the same, and it had a good diagnostic performance [10–12].

There are also come limitations in this study. This was a single-center study with a relatively small sample of children. Due to the exclusion of incomplete clinical and imaging data, the enrolled children were biased. We just focused on the correlation of prognosis and RALE score, did not combine, and compared with other clinical indicators.

## 5. Conclusion

RALE score based on CXR can be used in PARDS and has a better agreement among radiologist and pediatrician. Pulmonary complication and day 3 score whether greater than 21 points have a better discriminative effectiveness.

## Figures and Tables

**Table 1:** Comparison of survival and death groups.

	Survival (*n* = 72)	Death (*n* = 44)	Statistic	*P* value
Age (month)	12 (4, 54)	32 (8, 74)	*t* = −1.218	0.226
Male (%)	46 (63.9)	26 (59.0)	*χ* ^*2*^ = 0.267	0.605
Female (%)	26 (36.1)	18 (41.0)		
Infection (%)	40 (55.6)	25 (56.8)	*χ* ^*2*^ = 0.018	0.894
Intubation time (day)	6 (3, 15)	10 (3, 14)	*t* = 0.637	0.525
Pulmonary complication (%)	14 (19.5)	22 (50.0)	*χ* ^*2*^ = 11.913	**0.001**
Day 1 RALE score	28.21 ± 12.06	24.18 ± 12.31	*t* = 1.723	0.088
Day 2 RALE score	22.70 ± 10.84	27.00 ± 9.52	*t* = −2.155	**0.033**
Day 3 RALE score	20.43 ± 13.01	35.64 ± 11.22	*t* = −6.248	**<0.001**
Day 4 RALE score	18.57 ± 12.78	36.76 ± 7.89	*t* = −3.701	**<0.001**
Day 5 RALE score	16.93 ± 10.45	38.64 ± 10.18	*t* = −2.787	**<0.008**
OI	9.58 ± 5.73	14.34 ± 4.47	*t* = −2.227	**0.028**
SpO_2_ (%)	97.97 ± 0.93	95.34 ± 1.10	*t* *=* −7.782	**<0.001**
PEEP (cmH_2_O)	7.16 ± 3.19	10.84 ± 1.93	*t* = −7.602	**<0.001**

The bolded numbers are only to emphasize that the statistical results are significantly different.

**Table 2:** Comparison of infection and noninfection groups.

	Infection (*n* = 65)	Noninfection (*n* = 51)	Statistic	*P* value
Age (month)	38 (4, 87)	25 (8, 54)	*t* = −0.305	0.671
Intubation time (day)	5 (3, 15)	8 (3, 28)	*t* = −0.823	0.412
Pulmonary complication (%)	13 (20)	23 (45.1)	*χ* ^*2*^ = 8.714	**0.003**
Death (%)	25 (38.5)	19 (37.3)	*χ* ^*2*^ = 0.018	0.894
Day 1 score	24.20 ± 11.89	29.84 ± 12.10	*t* = −2.512	**0.014**
Day 2 score	25.13 ± 10.58	27.12 ± 10.63	*t* = −0.727	0.469
Day 3 score	25.92 ± 14.69	26.55 ± 14.08	*t* = −2.232	0.817
Day 4 score	33.00 ± 10.24	24.13 ± 9.13	*t* *=* *1.759*	0.102
Day 5 score	31.26 ± 10.36	24.16 ± 14.01	*t* *=* *1.767*	0.138
OI	12.51 ± 4.24	9.62 ± 4.17	*t* = 2.292	**0.027**
SpO_2_ (%)	97.32 ± 0.79	96.64 ± 0.87	*t* = 4.007	**<0.001**
PEEP (cmH_2_O)	10.96 ± 1.18	7.09 ± 2.78	*t* = 6.605	**<0.001**

The bolded numbers are only to emphasize that the statistical results are significantly different.

**Table 3:** RALE score comparison of survival and death groups in infection PARDS patients.

	Survive (*n* = 40)	Death (*n* = 25)	*t* value	*P* value
Day 1 score	23.90 ± 11.73	24.68 ± 12.38	−0.255	0.799
Day 2 score	20.50 ± 10.02	28.76 ± 9.57	−3.289	0.002
Day 3 score	19.15 ± 13.17	36.76 ± 9.74	−6.178	**<0.001**
Day 4 score	18.20 ± 13.25	37.50 ± 9.38	−3.584	**0.001**
Day 5 score	17.95 ± 9.15	38.76 ± 10.62	−4.005	**<0.001**

The bolded numbers are only to emphasize that the statistical results are significantly different.

## Data Availability

The data used to support the findings of this study are available from the corresponding author upon request.
